# Effects of Therapy with Light Emitting Diode (LED) in the Calcaneal Tendon Lesions of Rats: A Literature Review

**DOI:** 10.1155/2019/6043019

**Published:** 2019-02-03

**Authors:** Lízia Daniela e Silva Nascimento, Kárita Francisca e Silva Nascimento, Diego Rodrigues Pessoa, Renata Amadei Nicolau

**Affiliations:** ^1^State University of Piaui (UESPI), Health Sciences Center (CCS), Physical Therapy Course, Kinesiology, Biomechanics and Prosthesis and Orthotics, Teresina, Piauí, Brazil; ^2^PhD student in Biomedical Engineering, University of Vale do Paraíba (UNIVAP), São José dos Campos, São Paulo, Brazil; ^3^Master's Degree in Family Health, University Center UNINOVAFAPI, Teresina, Piauí, Brazil; ^4^Specialist in Physiotherapy in Re-Education of Motricity, University of Fortaleza (UNIFOR), Fortaleza, Ceará, Brazil; ^5^Physiotherapist, Municipal Health Foundation of Teresina, Piauí, Brazil; ^6^Speech and Hearing Therapist, Department of Social Assistance and Citizenship of the State of Piauí (SASC), Teresina, Piauí, Brazil; ^7^Specialist in Language, UNIFOR, Fortaleza, Ceará, Brazil; ^8^Bachelor in Physiotherapy, University Center of Santo Agostinho (UniFSA), Teresina, Piauí, Brazil; ^9^Master in Biomedical Engineering, UNIVAP, São José dos Campos, São Paulo, Brazil; ^10^Specialist in Teaching of Higher Education, UNINOVAFAPI University Center, Teresina, Piauí, Brazil; ^11^University of Vale do Paraíba, Department of Dentistry, Subjects of Bucomaxilofacial Surgery, Traumatology, and Stomatology, São José dos Campos, São Paulo, Brazil; ^12^Doctorate in Ciències Mèdiques Bàsiques, Universitat Rovira i Virgili, Tarragona, Spain; ^13^PhD and Masters in Biomedical Engineering-Laser Therapy, University of Vale do Paraíba, São José dos Campos, São Paulo, Brazil; ^14^Specialist in Research in Experimental Sciences Applied to Biomedicine, Universitat Rovira i Virgili, Tarragona, Spain; ^15^Specialist in Periodontics, Paulista Association of Dental Surgeons, São José dos Campos, São Paulo, Brazil

## Abstract

The objective of this review was to analyze original articles about the effects of therapy with LED in experimental models of calcaneal tendon lesions of rats. The search was performed in the period from February to May 2018, in the following electronic databases: MEDLINE, SciELO, and LILACS, besides the Google Scholar, using the descriptors “Achilles tendon”, “Rats”, “LED”, “Tendinopathy”, and “Low-level Light Therapy”, as well as their matching parts in the Portuguese and Spanish languages, related to and in association with the relevant terms to the content sought. From the descriptors used 215 works were found. After application of eligibility criteria 8 works were selected, in which positive results were found after the application of the LED. Regarding the main results found with phototherapy, we observed a significant reduction in inflammation. Only one article mentioned little reduction of inflammation. In relation to the number of sessions, there was wide variation, with an average of approximately 5 sessions every 24 hours. Studies in this review pointed out, therefore, positive results in the repair of the calcaneal tendon after therapy with irradiation LED; however, carrying out more experimental studies that help the standardization of parameters to be used in this therapy for further clinical studies becomes necessary.

## 1. Introduction

Tendinopathy is defined as a clinical presentation of pain leading to a reduction of the functional capacity and can be followed by the presence of signs as edema, pain, or thickening of the tendon. Many evidences have demonstrated that the lesion of the calcaneus tendon is the most common among athletes, besides being the most common target of spontaneous ruptures. The activities including running and jumping, such as badminton, volleyball, football, and athletics, are among those that have higher rate of injury in this structure, being the athletes of high level the most affected [1–3]. The calcaneal tendon lesion can occur in 9% of recreational runners and cause the end of the career of more than 5% of professional athletes [4].

The causes of acute or chronic lesions of the calcaneal tendon are usually related to mechanical and biological factors, alone or associated with a predominance of extrinsic factors in acute trauma. To be the Achilles tendon, a poorly vascularized tissue, which consequently has low nutrition and oxygenation, the capacity for tissue repair in this structure is low [5, 6].

The tendon tissue is a dense connective tissue, whose function is to transmit the force of a muscle to a bone. The tendons are constituted by fibroblasts and an extracellular matrix composed of fibrous proteins of collagen, elastin and proteoglycans, glycoproteins, and multiple saccharides. Collagen is the major structural protein and the main component of the extracellular matrix, representing about 86 to 95% of the wet weight [7]. The collagen Type I is considered responsible for the mechanical resistance of the tissue tendon and the collagen Type III has an important role in the healing process. The Type I collagen (thick fibers) is the primary collagen embedded in the structure of the tendon, and its increased production can improve tendon healing in cases of injury or rupture [8].

The tendon healing in acute lesions occurs in three stages: inflammation, proliferation, and remodeling. The inflammation happens to protect the body, eliminate harmful agents, and dilute the site and records an increase in capillary permeability and vasodilation, leading to the formation of edema. In the proliferation phase, there is an increase in the number and in the synthesis of fundamental substance and collagen. Already in the stage of remodeling the collagen fibers increase and there is a longitudinal realignment [9, 10].

Photobiomodulation (PBM) is a description of interventions with light therapy that modulates biological processes [11]. Research points that experimental studies have demonstrated evidence that the light emitting diode (LED), a subtype of the PBM, similar to low-intensity laser, can accelerate the process of tissue repair, once that is associated with the synthesis of adenosine triphosphate (ATP) and cellular proliferation [12]. Its mechanism of action involves the stimulation of fibroblast proliferation and increase in collagen synthesis, resulting in an improvement in the distensibility of the collagen and the resistance of the strains and tendon ruptures. In addition to these characteristics, the LED is a therapeutic resource for low cost, making it accessible to use on the occurrence of tendinous injuries [13, 14].

Some clinical studies [15, 16] and several experimental studies were performed using low-level light therapy (LLLT) in tendinopathies. From these researches, literature review studies have pointed out ideal irradiation parameters and respective therapeutic effects [17, 18]. However, there is only one study of this type with human beings on the use of LED therapy [19], and specifically on calcaneal tendon lesions, there was no* in vivo* study with human beings. Researches have been developed in animal models. Almost all studies use rats, because they are considered great experimental models of tendinopathies. Rats used in experiments are very similar to the human species both anatomically and physiologically [20], allowing reproducing induced lesions and allowing the histopathological assessment of the tendon [21].

Based on this context, the present study had as objective to perform a review of the literature on the effects of therapy with LED in experimental models (rats) of the Achilles tendon injuries, in order to facilitate and motivate the use of this therapy in clinical practice.

## 2. Method

The survey of the literature for this review was performed in the period from February to May 2018, using the following electronic databases: LILACS (*Latin American and Caribbean Center on Health Sciences Information*), MEDLINE (Online System for Search and Analysis of Medical Literature) via PubMed, and SciELO (*Scientific Electronic Library Online*), in addition to the Google Scholar. The descriptors used, after consulting the DeCS (Descriptors in Health Sciences) and MeSH (*Medical Subject Headings*), were“Tendão de Aquiles”, “Ratos”, “LED”, “Tendinopatia”, and “Terapia com luz de baixaintensidade”, as well as their synonyms in the English language, “Achilles tendon”, “Rats”, “LED”, “Tendinopathy”, and “Low-level light therapy”, and in the Spanish language, “Tendón de Aquiles”, “Ratones", “LED", “Tendinopatía", and “Terapia con luz de baja intensidad”, related to and in association with the relevant terms to the content sought.

There were elected as inclusion criteria: items available in their entirety, in Portuguese, English, or Spanish, with tendon injury heel approach induced in rats treated with LED in association or not to other therapies and published in the last 10 years, that is, from 2008 to 2018. Studies on LED therapy began in 2000 strongly supported by NASA [22]. From* in vitro* studies,* in vivo* studies began, justifying the one-decade time range chosen.

Incomplete articles were excluded from the study, which addressed the subject and not repeated articles. Review article is not found to list the Achilles tendon injury to use LED therapy. All review articles found on photobiomodulation (PBM) discussed the use of the low-level light therapy (LLLT) on calcaneal tendon, but not the LED therapy.

The articles were initially examined by title and abstract, in order to make sure that the study was performed with the use of LED, because many dealt with research carried out with laser. Then, the full texts of potential articles selected were evaluated.

## 3. Results

**Figure 1:**
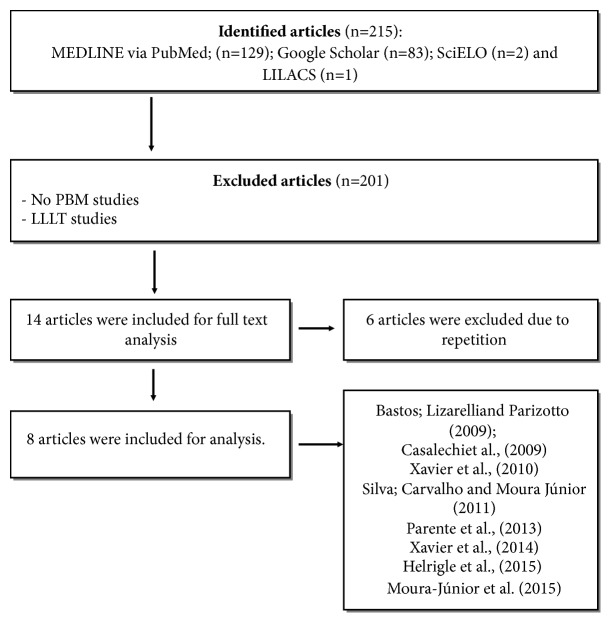
Selection flowchart of the studies used in the review of literature on the use of LED in tendon injuries.

Using the descriptors mentioned, 215 publications were obtained, being 129 in the MEDLINE, LiLacs 1, 83 in Google Scholar, and 2 in the SciELO. From these, 201 were excluded based on the title or the abstract. After analysis of the eligibility of the 14 articles those were selected for reading, 6 were excluded due to repetition. In this way, 8 studies were included in this review, all being carried out in Brazil (Figure 1 and Table 1).

In Table 1 the methodological characteristics are described and of the protocols used in the selected studies such as type of lesion, parameters of the instrument, and number of sessions, as well as the results obtained in each study after treatment. In all the studies analyzed positive effects were identified in the use of LED in the treatment of injuries of the Achilles tendons of rats. The work of Silva, Carvalho, and Moura Júnior (2011), however, mentioned that there was a significant result between the treated groups and the control groups of rats, stating that none of the animals verified the reconstruction of the normal structure of the tendon. None of the articles reported undesirable effects in the process of repair.

The experiments analyzed had their different wavelengths ranging from 625 to 945 nm. Two (25%) of these studies were carried out in a comparative manner between LEDs with different lengths of waves. In relation to the form of irradiation, five were performed in a timely manner at an angle of 90° and three were not reported in the form of application. The number of sessions described ranged from 3 to 21 days, with an average of 5 days in intervals of 48 hours (50%), power of 0.03W (50%), and average time of irradiation around 119 seconds among the studies that reported this parameter.

The type of histomorphometric analysis was predominant, alone or combined with another type of analysis. Of the studies, 2 (25%) underwent the histomorphometric analysis alone; 37.5% (3) used the histomorphometric analysis in conjunction with C-reactive protein (CRP), histological analysis, and Raman spectroscopy; 12.5% (1) performed histological analysis only; 12.5% (1) performed CRP isolated; and 12.55% (1) underwent analysis using the Elisa test. It was observed, however, that none of the studies presented the time (days) of analysis.

The most significant results obtained with the therapy decreased inflammatory response by reducing of cells and modulation of inflammatory mediators, in addition to the increase of fibroblasts, collagen synthesis, and reorganization. Some of the other results mentioned were reduction of interleukins (IL-6 and IL-10).

## 4. Discussion

The objective of this study was to perform a review of the literature about the experimental studies that investigated the healing process in the calcaneal tendon lesions with the use of LED. In general, the majority of the studies reviewed in this review article demonstrated satisfactory results regarding the use of low-intensity LED therapy on the process of tissue repair, modification of the cell behavior, and the vascular formation, as well as the production of collagen, fibroblasts, and epithelial tissue in rats tenotomized [24–28].

Currently, the LED therapy has been an alternative clinical modality used in several experimental studies in relation to LLLT, because it is a feature with lower cost to lasers, which present similar results to LLLT, due to its PBM effects that occur soon after the stimulation of photoreceptors found in mitochondrial crests (cytochrome c oxidase), which favor angiogenesis, the increase in the synthesis of ATP, the increment in the increase in the rate of production of fibroblasts and collagen synthesis, the decrease in oxidative stress, and the keeping tissue homeostasis [28–31].

Casalechi et al. [23] performed a study that aimed to evaluate the effects of therapy LED on the process of tissue repair in rats submitted to experimental traumatic process of tendinopathy; a significant reduction in the number of fibroblasts was evidenced; after irradiation with the therapy LED, findings that reinforce the results were obtained by Parente et al. [13]; they concluded in their study that the therapy LED propitiated beneficial effects on the reduction in the number of inflammatory cells and the alignment of collagen fibers, which corroborates with the findings of Silva; Carvalho and Moura-Junior [25] observed effects PBM after irradiation with a dose equal to 4 J/cm^2^, favoring a higher proinflammatory action.

Bastos et al. [14] compared the effects between the irradiation with different wave lengths between the LED therapy (630 nm (visible) and 880 nm (invisible) and the lilt (685 nm (visible) and 830 nm (invisible) in rats submitted to tenotomy process of the Achilles tendon; it was established at the end of the study that the use of therapies in the region of the electromagnetic spectrum of near infrared (880 nm and 830 nm) favored the improvement of the organization, aggregation, and alignment of the collagen, thus contributing to a more effective tissue repair. Already Moura Junior et al. [28] evaluated the effects of treatment made by therapeutic ultrasound (US) and the LED therapy (625 nm and 945 nm) in an experimental model of tenotomy of the Achilles tendon; it has been proven that the US has promoted the reduction of inflammatory cells in different experimental times (7^th^ and 14^th^ day), has already LED therapy (625 nm), and showed greater synthesis of collagen type I (14^th^ day) in relation to the other treatments.

Ferreira et al. [31] stated that the electromagnetic radiation, coherent and noncoherent, favors significantly neovascularization of the injured tissue, collaborating with the healing process, reducing the necrozed area, through the increase of blood flow in the places of the lesion; moreover, it contributes to the reduction in the number of inflammatory cells and promotes stimulating effect on alpha tumor growth factor (TGF-*α*) and platelet-derived growth factor (PDGF), the latter being directly related to the tissue repair process.

Proposed studies described that LED therapy is able to modulate the expression of the gene RNAm in proinflammatory mediators in an experimental model of tendonitis [24, 26]. Xavier et al. [24] investigated the effects of therapy LED (880±10 nm) in an experimental model of Achilles tendonitis induced by collagenase. It has been proven that the therapy LED promoted a decrease in the influx of inflammatory cells, expression of RNAm, IL-1 beta, IL-6, and TGF-*α* and cyclooxygenase-2 (COX-2). Xavier et al. [26] evaluated the quantitative alterations in the composition of collagen (Types I and III) in tendons after tenotomy and employment of LED therapy. It kwas possible to conclude that the therapy LED (880 nm) is showed to be effective in increasing the expression of RNAm, IL-10, and collagen (Types I and III), suggesting that the appeal has potentially therapeutic effects in the calcaneal tendon lesions.

Helrigle et al. [27] showed that the therapy of low-intensity LED was favorable to the modulation of IL-6, IL-10, and TNF-*α* by suppressing the proinflammatory action, through the determination of eicosanoids on the inflammation of the tendon. The efficiency of the LED reported above may be related to different effects of the LED on the dosimetric parameters (energy, wave length, time, and power), constituting one of the most important characteristics in the protocol of irradiation, a time at which photobiomodular effects are important in the process of tissue repair.

In this study and the articles searched for review, we found improvement of the quality of the repair of the calcaneus tendon of rats. Postoperative histological assessment and the use of the LED on the Achilles tendon, however, are necessary to standardize between dosimetric parameters (energy, wave length, time, and power) and the mechanisms that influence functional recovery of the tendons repaired with LED, since the establishment of appropriate protocols can help more quickly the incorporation of this type of phototherapy in clinical practice in orthopedics, traumatology, and sports medicine.

## 5. Conclusion

The LED therapy gave positive results in lesions of the calcaneus tendon; however, there is not a consensus regarding the parameters to be used. Because of the different protocols described, it is important to undertake more studies that help to standardize the parameters of this therapy applied to lesions in the calcaneal tendon.

## Figures and Tables

**Table 1:** Articles included in the review, organized in chronological order of publication: authors, years, type of lesion, lesion area, LED parameters, irradiation form, number of sessions, moment of sessions, type of analysis, time of analysis, and results.

Authors/Year	Type of lesion	LED Parameters	Irradiation form	N. of sessions	Moment of sessions	Type of analysis	Time of analysis (days)	Results
Bastos; Lizarelli; Parizotto (2009) [14]	Partial tenotomy	ʎ (nm) 880 and 630 P (W): 0.025 Beam area (cm^2^): 0.28 T (s): NI ED (J/cm^2^): 6 E (J): NI PD(W/cm^2^):NI	NI	5 and 10	Every 24 hours	HM	NI	After 5 days: ↑ proliferation of fibroblasts. After 10 days: ↑ alignment and organization of collagen fibers.

Casalechi (2009) [23]	Tenotomy	ʎ (nm) 640 P (W): 0.1 Beam area (cm^2^): 0.5 T (s): 120 ED (J/cm^2^): 20 E (J): NI PD (W/cm^2^): NI	90° contact	6.13 and 20	Beginning 1h after tenotomy and repetition every 24h	HM	NI	↓ inflammatory cells ↓remodeling; ↑ organization of collagen fibers.

Xavier et al (2010) [24]	Tendinitis	ʎ (nm) 880 P (W): 0.022 Beam area(cm^2^): 0.5 T (s): 170 D/E (J/cm^2^): 7.5 E (J): NI PD(W/cm^2^): NI	90° contact	4 and 7	Beginning 12h after lesion and repetition every 48h	HM and CRP	NI	↑ inflammatory cells; re-organization of collagen fibers; ↓ mRNA, IL-6 and TNF-a expression.

Silva; Carvalho; Moura Júnior (2011) [25]	Tendinitis	ʎ (nm) 640 P (W): 0.03 Beam area (cm^2^): 0.5 T (s): 120 ED (J/cm^2^): 4 E (J): NI PD(W/cm^2^): NI	90° contact	6 and 13	Every 24 hours	HT and HM	NI	After 7 and 14 days: ↑ fibroblasts when compared to groups with tendinitis; Lack of significant result between groups of treated and healthy rats Without re-construction of the normal structure of the tendon in all animals.

Parente et al (2013) [13]	Tendinopathy	ʎ (nm): 640 P (W): 0.096 Beam area (cm^2^): 3.3 T (s): 70 ED (J/cm^2^): 2.04 E (J): NI PD(W/cm^2^): NI	NC	3 and 10	Beginning 12h after lesion and repetition every 48h	HT	NI	TEND groups after 7 and 21 days: ↑ inflammatory cells ↑disorganization of collagen fibers. LED groups after 7 and 21 days: amount of cells and organization of collagen fibers similar to control groups. DROG groups 7 and 21 days: number of cells similar to TEND groups with ↑ organization of collagen fibers.

Xavier et al(2014) [26]	Tendinitis	ʎ (nm): 880 P (W): 0.02 Beam area (cm^2^): 0.5 T (s): 170 D/E (J/cm^2^): 7.5 E (J): NI PD(W/cm^2^): NI	NC	4 e 7	Beginning 12h after lesion and repetition every 48h	CRP	NI	After 7 days: ↑ IL -10. After 7 and 14 days: ↑ types I and III collagen

Helrigle et al (2015) [27]	Tendinopathy	ʎ (nm): 945 P (W): 0,032 Beam area (cm^2^): 1 T (s): 120 ED (J/cm^2^): 3.84 E (J): 3.84 PD(W/cm^2^): 0,032	90° contact	2, 4 and 7	Beginning 12h after trauma and repetition every 48h	Immunoenzymatic test (ELISA)	NI	After 7 and 14 days: ↓ IL-6 and TNF-*α*, ↑ IL-10; ↓ inflammatory effects.

Moura Júnior et al (2015) [28]	Tenotomy	ʎ (nm) 625 e 945 P (W): 0.033 Beam area (cm^2^): 0.5 T (s): 60 ED (J/cm^2^): 4 E (J): 2 PD(W/cm^2^): 0.5	90° contact	6 and 13	Beginning 24h after surgery	HT, HM and RS	NI	LED 945 groups after 7 days: ↓ inflammatory cells; ↑ type-III collagen (Raman); LED 625 groups after 7 days: no significant difference of the control group; ↑synthesis of type-I collagen (Raman).

ʎ: wavelength, ED: energy density, E: energy, PD: power density, T: time, NI: no information, CRP: C-reactive protein, HT: histological analysis, HM: histomorphometric analysis, and RS: Raman spectroscopy.
